# The Effect of Organizational Changes on the Psychosocial Work Environment: Changes in Psychological and Social Working Conditions Following Organizational Changes

**DOI:** 10.3389/fpsyg.2019.02845

**Published:** 2019-12-20

**Authors:** Lise Fløvik, Stein Knardahl, Jan Olav Christensen

**Affiliations:** Department of Work Psychology and Physiology, National Institute of Occupational Health (STAMI), Oslo, Norway

**Keywords:** organizational change, psychosocial work environment, occupational health, mental distress, sick leave, leadership

## Abstract

**Purpose:** The present study aimed to clarify the prospective effects of various types and frequencies of organizational changes on aspects in the psychosocial work environment.

**Methods:** The study had a prospective, full-panel, repeated measures design. Data were collected by self-administered, online questionnaires, with a 2-year interval between measurement occasions. Five types of organizational change were assessed – company restructuring, downsizing, layoffs, partial closure, and partial outsourcing. The effects of change on eleven, specific work factors were measured utilizing QPS Nordic. At baseline, 12652 employees participated, while 8965 responded at follow-up. Generalized estimating equations were utilized to estimate the effects of change taking place within the last 12 months or more than 24 months prior.

**Results:** Cross-sectional analyses, i.e., changes occurring within the last 12 months, showed all 11 work factors to be statistically significantly associated with the organizational changes restructuring, downsizing, and partial closure (coefficients ranging −0.28 to 0.04). In the prospective analyses, i.e., the effects of change taking place more than 24 months prior, associations were no longer significant for a number of work factors, although all types of organizational change remained significantly associated with at least three work factors (coefficients ranging −0.14 to 0.05). Following repeated organizational changes, statistically significant associations were shown for all 11 work factors (coefficients ranging from 0.39 to −0.04).

**Conclusion:** Following both separate and repeated organizational change, various psychological and social work factors were altered, with the most pronounced effects following repeated change. These results suggest the implementing organizational change, especially repeated change, may have an adverse effect on various parts of the psychosocial work environment. The negative effects of a company’s psychosocial working conditions may contribute to the adverse health effects often observed following such changes and help explain why many change initiatives fail to reach its intended results.

## Introduction

Organizational change has repeatedly been associated with adverse effects on employee health (Oreg et al., 2011). Large-scale organizational changes, such as company restructuring, downsizing and outsourcing have been linked to somatic and mental health complaints, presenteeism and long-term sick leave (Kivimäki et al., 2001; Bamberger et al., 2012). However, a thorough understanding of why organizational changes are associated with adverse health effects is still pending. Clarifying the repercussions of organizational change for workplaces and employees is an essential first step to preventing adverse health effects of and facilitating healthy, successful change. Prior meta-analytic studies have shown a wide range of psychological and social work factors, such as leadership, role conflict and ambiguity, job demands, control and job insecurity to predict employee well-being, health and sick leave (Viswesvaran et al., 1999; Stansfeld and Candy, 2006; Nahrgang et al., 2011; Lang et al., 2012; Schyns and Schilling, 2013; Virtanen et al., 2013; Schmidt et al., 2014; Theorell et al., 2015), as well as the change process and end-result (Schuler and Jackson, 2001; Hoag et al., 2002). In order to elucidate whether extensive company change influence central aspects of the psychosocial work environment the present study aimed to clarify the effects of various types of organizational changes, separately and co-occurring as well as repeated over time, on 11 specific psychological and social work factors.

“Organizational change” pertains to the altering of structures, strategies, procedures or cultures of organizations (Quattrone and Hopper, 2001). The term encompasses both the process by which this happens (i.e., “how”) and the content of what is being altered (i.e., “what”). By definition, change implies a shift in the organization from one state to another. This shift may be deliberate, with the aim of gaining or losing specific features of the organization to attain a defined goal, or it may be less deliberate, perhaps occurring as a consequence of developments outside the control of the organization. Moreover, during the change process, additional parts of the organization may be unintentionally affected, particularly when change is experienced as excessive (Stensaker et al., 2001). Such unintended repercussions of organizational change may be both positive and negative (Jian, 2007), and may be more likely when a large number of transactions are required to implement the change decision and many specialized problem-solving capabilities are invoked (Casa and Lodge, 2015). Either way, organizational change represents something novel and intrinsically unknown and uncertain for the organization and its members, which may disrupt existing structures and processes. Thus, organizational change can be experienced both as an opportunity to gain and as a risk of losing and may involve redesign of tasks and responsibilities that alter existing work content and –environment in various foreseen and unforeseen ways. Nevertheless, while prior studies have linked organizational change to somatic and mental health (Vahtera et al., 1997; Kivimäki et al., 2001, 2003; Probst, 2003; Moore et al., 2004; Vahtera et al., 2004), less is known about repercussions of organizational change for psychosocial working conditions that are relevant to health.

A psychosocial work environment consists of organizational-, social-, and psychological factors which govern and define the content and quality of various aspects of work (Nieuwenhuijsen et al., 2010). *Organizational* work factors include formal and structural conditions that regulate how work is carried out, e.g., employment contracts and work schedules. *Social* work factors comprise the relational aspects of a workplace, such as social climate, support from superiors and co-workers. *Psychological* work factors refer to individual-level aspects of work, such as perceived levels of autonomy, job demands and predictability. Prior studies have linked an organization’s psychosocial working conditions to both employee- and organizational outcomes (Stansfeld and Candy, 2006; Holden et al., 2011), such as worker health (Lau and Knardahl, 2008; Väänänen et al., 2008; Bambra et al., 2009; Häusser et al., 2010; Nahrgang et al., 2011; Schmidt et al., 2014; Read and Laschinger, 2015; Schmidt et al., 2018), sick leave (Head et al., 2006) and company productivity (Dollard and Neser, 2013; Dysvik and Kuvaas, 2013; Poulsen et al., 2016; Montano et al., 2017). Despite the aforementioned awareness of the potential of organizational change to upset various organizational systems as well as employee health, few studies seem to have assessed effects of organization change on specific factors in the psychosocial work environment that are known to be associated with health. The present study assessed the effect of various types and frequencies of organizational change on 11 distinct work factors pertaining to *job tasks* (job control, job demands), *job roles* (role clarity, role conflict), *leadership* (fair-, empowering-, supportive leadership), *social aspects* (support from co-workers, social climate) and *predictability* (job predictability, future employability).

During change implementation, the organization attends to various change-related tasks in addition to the ordinary, day-to-day activities. In sum, this may increase the total amount of work and job tasks employees are faced with (Kivimäki et al., 2001). The need for management to exert control in the planning and implementation process may also leave less room for employees to influence their own job to the same extent as before, and may thus affect employees’ experience of their own job control during the process (Paulsen et al., 2005). Hence, organizational change may be associated with increased job demands (i.e., the amount of work and time demands) and a decrease of job control (i.e., influence over decisions regarding one’s tasks, co-workers and clients). When major shifts take place within an organization, the rearrangement of employee roles and responsibilities are often a central part of the process. Such rearrangement may result in employees facing conflicting demands, lack of resources to complete one’s additional assigned tasks or uncertainty related to the objectives and expectations of one’s new role (Baillien and De Witte, 2009). Thus, large-scaled workplace changes could increase employee’s sense of role conflict (i.e., conflicting demands and lack of resources) and lower the sense of role clarity (i.e., clarity regarding a roles responsibilities and expectations). The need for management to invoke tough and sometimes unpopular decisions, e.g., in a downsizing or layoff process, may also affect employees perception of management and superiors following organizational changes (Gilley et al., 2009; Holten and Brenner, 2015; Neves and Schyns, 2018). When the consequences of change involve the potential loss of valued aspects such as specific tasks, collegial relationships or the very existence of one’s job, one may surmise that employee perception of management as just or fair could be affected. Moreover, changes initiated by external forces, e.g., market demands or technological innovation, and invoked by management may also leave less room for including employees in decision making and planning. Exercising an inclusive and empowering leadership style may thus be challenging during change implementation. The added demands given to managers in this process may also leave fewer resources and room for superiors to provide the support and attentiveness they normally are able to give their employees, which may affect employee’s perception of management as supportive. Hence, organizational change may lead to a decrease in employee perception of leadership as fair (i.e., equal treatment of employees), empowering (i.e., including) and supportive (i.e., attentive and present). The relational aspects of the organizational may also be affected during extensive workplace changes, as the collegial composition may be rearranged or colleagues have to compete over new or remaining positions. As a result, social cohesion within the group may deteriorate and collegial support diminishes (Campbell and Pepper, 2007). Thus, organizational change may be associated with a decrease in perceived support from co-workers and social climate as inclusive and trusting. Employees’ sense of predictability regarding both present and future job prospects could also be affected by exposure to extensive workplace changes (Probst, 2003; Baillien and De Witte, 2009). Change naturally involves some degree of uncertainty regarding the outcome and future. As extensive organizational changes often are management-driven with little employee involvement, uncertainty may be extra prominent. Furthermore, organizational change is often driven by changes in external factors such as globalization, market demands or technological innovation, which makes predicting the future jobs even more complex. Hence, organizational changes may be associated with a decrease in employee perception of short-term job predictability and future employability.

As the rate of organizational change is increasing, a larger part of the workforce is likely to experience multiple changes or repeated organizational changes during their careers, some of which they may deem excessive. To our knowledge, a limited number of studies have examined how exposure to repeated organizational changes influence specific factors in the work environment (Moore et al., 2004). These studies have reported stronger effects following repeated change than separate change, but only on outcomes such as employee health and sick leave (Isaksson et al., 2002; Moore et al., 2004; Wagstaff et al., 2016). Thus, one may speculate whether implementing multiple, repeated changes may also be associated with a stronger effect on psychosocial work factors than single change efforts (Klarner et al., 2011).

In developing targeted interventions aimed at reducing the potential adverse effects of organizational change on employee health, identifying the underlying mechanisms in this stressor-strain relationship is an imperative first step. Interventions aimed at reducing or alleviating the effect of risk factors in the work environment have shown the potential of such interventions to reduce depressive symptoms and absenteeism and to improve productivity both during and following organizational changes (Bambra et al., 2009; Kelloway and Barling, 2010; Houtman and Lourijsen, 2012). The effect of organizational change on specific factors in the work environment may represent such a mechanism in which the work factors may either moderate or mediate the relationship between change and health. In order to illuminate the effect of various specific types of organizational change as well as repeated change on central aspects of the psychosocial work environment, the current study examined both the cross-sectional and prospective associations of separate and repeated organizational change with 11 specific psychological and social work factors.

## Materials and Methods

### Study Design

The study was a part of the project “The New Workplace: work, health and participation in working life” initiated and carried out by the Norwegian National Institute of Occupational Health (STAMI). The project was conducted in line with the World Medical Association Declaration of Helsinki, and approved by the Data Inspectorate of Norway and the Norwegian Committee for Medical and Health Research Ethics, Region South East (REC). The study had a prospective, full panel study design, with data collected with a 2-year interval. Baseline data collected between 2004 and 2013, with follow-up 2 years later, respectively. All data were collected by a self-administered, online questionnaire. The participating organizations either contacted STAMI directly as a response to an invitation to participate in the study posted on the institute’s webpages, or on requesting assistance in a general work environment survey.

### Subjects

A total of 66 Norwegian organizations participated in the study, representing both public and private sector and a variety of professions, company sizes and sectors.

Upon accepting to participate, information regarding the project was initially given at the company level. All current employees were invited to participate in the study and received an information letter by postal mail, containing a unique ID-code for accessing the online questionnaire. Respondents were allotted time during work hours to complete the questionnaire but also had the opportunity to complete the questionnaire at home. Respondents had the opportunity to log in an unlimited number of times to access to complete the questionnaire. Inclusion criteria for both the cross-sectional and prospective sample were completing all items for each individual work factor at both T1 and T2.

### Predictor: Organizational Change

#### Specific Organizational Change

We assessed the effects of five distinct types of organizational changes. These were company restructuring, downsizing, layoffs, partial closure, and partial outsourcing. To clarify, “downsizing” refers to a temporary termination of job contract with the chance of rehiring, while “layoffs” refers to permanent termination of the job contract. Each type of change was assessed by a single item with a dichotomous response (“yes”/”no”) and inquired whether the organization in which the employee worked had implemented a specific type of change within the last 12 months. Examples: “During the last 12 months has your company undergone restructuring?,” and “During the last 12 months has your company undergone downsizing?”

#### Multiple Organizational Changes

To assess the effect of multiple organizational changes occurring simultaneously, a three-category predictor variable was created based on the five change items. The categorical predictor demonstrated whether employees had experienced (i) “No type of organizational change at baseline,” (ii) “One type of organizational change at baseline,” or (iii) “Two or more types of organizational change at baseline.”

#### Repeated Organizational Change

To assess the effects of repeated organizational changes, a four-category predictor variable was created based on the five change items. The predictor demonstrated whether employees had experiences (i) “No type of change at baseline or follow-up,” (ii) “At least one type of change at baseline, but none at follow-up,” (iii) “At least one type of change at follow-up, but none at baseline,” or (iv) “At least one type of change at baseline *and* at least one type of change at follow-up.”

### Outcome: Psychological and Social Work Factors

The psychological and social work factors were measured by the General Nordic Questionnaire for Psychological and Social Factors at Work (QPS_Nordic_) (Ørhede et al., 2000; Wännström et al., 2009). QPS_Nordic_ is a validated questionnaire designed to assess a comprehensive set of social and psychological aspects in the workplace. The effects of organizational changes on 11, specific work factors were assessed. These were six psychological work factors (job control, job demands, job predictability, perceived future employability, role clarity, and role conflict) and five social work factors (empowering leadership, fair leadership, social climate, support from co-worker and support from superior). Each factor was measured by multiple items, ranging from two to five items depending on the factor. Responses on all items were given on a five-point Likert scale, ranging from “1 = very seldom or never” to “5 = very often or always.” For each work factor, a mean score was calculated. For all work factors, Cronbach’s α was calculated at baseline and follow-up and were within the range of 0.71 (“role conflict“) to 0.88 and (“empowering leadership”).

### Confounders

All analyses included the variables age, sex, skill level, and place of employment (private vs. public organizations) as potential confounders. Age was divided into three age groups, (i) “<35,” (ii) “35–55,” and (iii) “>55.” Skill level was divided into five categories reflecting years of formal education required in various professions. The categorization was done using the Standard Classification of Occupations (STYRK), which is based on the International Standard Classification of Occupations (ISCO-88) and developed by Statistics Norway (SSB). The five skill level categories were: (i) “<10 years of education,” (ii) “10–12 years of education,” (iii) “13–15 years of education,” (iv) “>15 years of education,” and (v) “Unspecified,” which included occupations requiring no formal education.

### Statistical Analyses

#### Generalized Estimating Equations

The cross-sectional and prospective associations between the separate types and frequencies of organizational changes and the various work factors were estimated utilizing linear regression analyses by the Generalized Estimated Equations method (GEE). The method is based on the generalized linear model and allows for the analyses of correlated observations, such as repeated measures or clustered data. In addition, the method allows for samples to have a non-normal error distribution on the response variable. The GEE approach was chosen as it accounts for the potential correlated responses within sample clusters, which fit the present data well as it was clustered within organizations (Zorn, 2001; Hubbard et al., 2010). The GEE method gives a population parameter estimate based on the average of clusters in the data (Hardin and Hilbe, 2002; Hanley et al., 2003). Hence, the GEE method estimates the average response in a population-based on the average of clusters within a sample. The GEE analysis provides the option to predefine the anticipated correlation structure in the data, for instance independent, autoregressive, compound symmetry, or unstructured. In the present analyses, the unstructured option was chosen since no theoretical grounds were present to expect a specific correlation structure in the data. In addition, the unstructured option does not impose any constraints in the correlation structure in the analyses (Hardin and Hilbe, 2002). GEE has previously been widely applied in epidemiological studies where data have been correlated as the method may handle various types of prior, unidentified correlations between measurements (Merlo, 2003; Skrondal and Rabe-Hesketh, 2003; Cui and Qian, 2007). All analyses were run using IBM SPSS Statistics, version 24.0 (IBM, Armonk, NY, United States), with the level of statistical significance set to *p* < 0.05.

#### Cross-Sectional Analyses

In the cross-sectional analyses pertaining to specific, separate organizational changes, we ran both uni- and multi-variate regressions separately with each type of change as predictor and each type of work factor as outcome. In the analyses pertaining to the effects of multiple changes, we utilized the aforementioned three-category variable as predictor and ran the analyses for each work factor separately.

#### Prospective Analyses

In the prospective analyses, both uni- and multi-variate regressions were run separately with each type of change as predictor and each type of work factor as outcome. The analyses were run in two steps. In the first step, Model I, analyses were adjusted for age, sex, skill level and place of employment, while in step two, Model II, analyses were also adjusted for baseline levels of the work factor in question. In the analyses pertaining to multiple changes, we ran simple regressions with the three-level categorical predictor variable for each work factor separately. As in the analyses pertaining to specific changes, all analyses were conducted in two steps. Lastly, in the analyses pertaining to the effects of repeated change, we ran simple regressions with the aforementioned four-level categorical predictor for each work factor separately. These analyses were also conducted in two-steps.

## Results

### Baseline Characteristics

The mean age at baseline was 44.34 (*SD*: 10.5). Of the included subjects 20.9% were under the age of 35, 61.9% were between the age of 35–55, while 17.2% were older than 55. Women constituted 54.7% of the sample. Skill level at baseline was as follows: >15 years of formal education 26.9%, 13–15 years 24.7%, 10–12 years 38.7%, >10 years 1.0%, and Unspecified 8.7%. For further details, see Table 1.

**TABLE 1:** Sample characteristics.

	**Invited subjects**		**Baseline sample**				**Prospective sample**			
	***N***	**%**	***N***	**%**	**Mean**	***SD***	***N***	**%**	**Mean**	***SD***
**Sex**										
Female	8467	54.7	6478	51.2			4733	52.8		
Male	6998	45.3	6174	48.8			4232	47.2		
Total	15465		12652	100			8965			
Missing			2841	18.3						
**Age**										
>35			2638	20.9			1740	19.4		
35–55			7837	61.9			5742	64.0		
>55			2177	17.2			1483	16.5		
Total			12652	100	44.34	10.54	8965	100		
**Skill level**										
>15			3408	26.9			2438	27.2		
13–15			3126	24.7			2334	26.0		
10–12			4895	38.7			3214	35.9		
<10			127	1.0			71	0.8		
Unspecified			1096	8.7			908	10.1		
**Workplace**										
Public sector	11792	76.2	9914	78.4			6995	78.0		
Private sector	3673	23.8	2738	21.6			1970	22.0		
**Organizational change**										
No change			3445	36.1			3780	43.0		
One change			3474	36.4			2740	31.2		
Two or more changes			2643	27.7			2268	25.8		
**Organizational change**										
Reorganization			5356	55.0			4448	49.8		
Downsizing			2406	15.5			1945	21.8		
Layoffs			911	5.9			1081	12.1		
Partial closure			1238	8.0			1062	11.9		
Partial outsourcing			774	5.0			994	11.2		

*Characteristics of baseline sample and prospective sample.*

### Non-response Analysis

Women were less likely to be non-respondents (OR 0.72, 95% CI 0.66–0.78), along with employees aged 35–55 years (OR 0.82, 95% CI 0.74–0.91). Respondents employed in private sector companies were also less likely to be non-respondents (OR 0.86, 95% CI 0.78–0.95). As for skill level, respondents employed in jobs requiring 10–12 (OR 1.35, 95% CI 1.22–1.49) and <10 years of formal education (OR 1.84, 95% CI 1.26–2.67) were also more likely to be non-respondents. For further details, see Table 2.

**TABLE 2:** Non-response and attrition analyses.

	**Non-response analysis**				**Attrition analysis**			
	***N***	**%**	**OR**	**95% CI**	***N***	**%**	**OR**	**95% CI**
**Sex**								
Female	6478	51.2			4733	52.8	1.09	0.99–1.20
Male	6174	48.8	–	–	4232	47.2	–	–
Total	12652	100.0			8965	100		
**Age**								
<35	2638	20.9	–	–	1740	19.4	–	–
35–55	7837	61.9	**0.82**	**0.74–0.91**	5742	64.0	**0.78**	**0.69–0.87**
>55	2177	17.2	1.10	0.97–1.26	1483	16.5	0.89	0.76–1.04
**Skill level**								
>15	3408	26.9	–		2438	27.2	–	–
13–15	3126	24.7	**0.82**	**0.73–0.92**	2334	26.0	**1.55**	**1.36–1.77**
10–12	4895	38.7	**1.35**	**1.22–1.49**	3214	35.9	**1.65**	**1.45–1.87**
<10	127	1.0	**1.84**	**1.26–2.67**	71	0.8	**1.86**	**1.13–3.08**
Uspesifisert	7096	8.7	**0.53**	**0.43–0.62**	908	10.1	0.80	0.65–0.98
**Workplace**								
Public sector	9914	78.4	–	–	6995	78.0	–	–
Private sector	2738	21.6	**0.86**	**0.78–0.95**	1790	22.0	**1.19**	**1.07–1.33**
**Organizational change**								
No change					3184	35.5	–	–
One change					3310	36.9	1.05	0.94–1.18
Two or more changes					2471	27.6	1.09	0.97–1.23

*Non-response analysis and attrition analysis. Non-response defined as not completing work factor items at baseline. Attrition defined as completing work factor items baseline, but not at follow-up. The bold significance values are p < 0.05.*

### Sample Attrition

Being employed in private sector was linked to dropout at follow-up (OR 1.19, 95% CI 1.07–1.33). Working in an occupation requiring 13–15 years of formal education (OR 1.55, 95% CI 1.36–1.77), 10–12 years (OR 1.65, 95% CI 1.45–1.87) or less than 10 years of formal qualifications (OR 1.86, 95% CI 1.13–3.08) were also associated with not participating at follow-up. On the other hand, employees aged 35–55 were negatively associated with dropout (OR 0.78, 95% CI 0.69–0.87). Gender was not associated with attrition. For further details, see Table 2.

### Effects of Separate Organizational Change

For a short summary of all associations, see Table 3.

**TABLE 3:** Separate organizational change.

	**Change < 12 months**					**Change > 24 month ago**				
	**Reorganization**	**Downsizing**	**Layoffs**	**Partial closure**	**Partial outsourcing**	**Reorganization**	**Downsizing**	**Layoffs**	**Partial closure**	**Partial outsourcing**
Empowering leadership	^∗∗∗^	^∗∗∗^	^∗∗∗^	^∗∗∗^	^∗∗∗^	ns	ns	^∗^	ns	ns
Fair leadership	^∗∗∗^	^∗∗∗^	^∗^	^∗^	^∗∗^	ns	ns	^∗^	^∗∗^	^∗^
Job control	^∗∗∗^	^∗∗∗^	^∗∗^	^∗∗^	ns	ns	ns	ns	ns	ns
Job demands	^∗∗∗^	^∗∗∗^	^∗∗∗^	^∗∗^	^∗∗∗^	^∗∗^	^∗^	ns	^∗∗^	ns
Role clarity	^∗∗∗^	^∗∗∗^	ns	^∗∗∗^	^∗^	ns	^∗^	ns	^∗∗^	ns
Role conflict	^∗∗∗^	^∗∗∗^	^∗∗∗^	^∗∗∗^	^∗∗^	^∗∗∗^	ns	^∗∗∗^	ns	ns
Social climate	^∗∗∗^	^∗∗∗^	^∗∗∗^	^∗∗∗^	^∗∗∗^	^∗∗∗^	ns	^∗∗^	ns	ns
Support superior	^∗∗∗^	^∗∗∗^	^∗∗∗^	^∗∗∗^	^∗∗∗^	ns	ns	ns	ns	ns
Support co-worker	^∗∗∗^	^∗∗∗^	^∗∗∗^	^∗∗∗^	^∗∗∗^	ns	ns	ns	ns	ns
Job predictability	^∗∗∗^	^∗∗∗^	^∗∗∗^	^∗∗∗^	^∗∗∗^	^∗∗^	^∗∗^	^∗∗^	^∗∗∗^	^∗∗∗^
Future employability	^∗∗∗^	^∗∗∗^	^∗∗∗^	^∗∗∗^	^∗∗∗^	^∗^	^∗^	^∗^	ns	^∗∗^

*Summary of univariate analyses, “adjusted own effect”: cross-sectional and prospective analyses. Separate, linear regressions with separate, organizational change at baseline as predictor and work factor as outcome. Change < 12 months. Adjusted for age, sex, skill level, and place of employment. Change > 24 months (Model II). Adjusted for age, sex, skill level, place of employment, and work factor at baseline. ^∗^*p* < 0.001, ^∗∗^*p* < 0.01, ^∗∗∗^*p* < 0.05.*

#### Cross-Sectional Analyses

##### Univariate

The analyses of change reported to have occurred during the last 12 months prior to baseline showed company restructuring, downsizing, and partial closure to be statistically significantly associated with all work factors, with *b-*values ranging from −0.28 to 0.04. Layoffs and partial outsourcing were also statistically significantly associated with most work factors, with the exception of job control, which was not statistically significantly related to partial outsourcing, and role clarity, which was not statistically significantly related to layoffs. See Table 4 for further details.

**TABLE 4:** Separate organizational change.

		**Change <12 months prior**			**Change >24 months prior**					
		**Model I**			**Model I**			**Model II**		
		***B***	***p*-Value**	**95% CI**	***B***	***p*-Value**	**95% CI**	***B***	***p*-Value**	**95% CI**
Empowering leadership	Reorganization	**−0.09**	**0.000**	**0.87 to 0.96**	**−0.08**	**0.006**	**−0.13 to −0.02**	**−**0.02	0.537	**−**0.06 to 0.03
	Downsizing	**−0.15**	**0.000**	**0.82 to 0.91**	**−0.09**	**0.009**	**−0.15 to −0.02**	**−**0.02	0.578	**−**0.07 to 0.04
	Layoffs	**−0.22**	**0.000**	**0.74 to 0.87**	**−0.19**	**0.000**	**−0.29 to −0.09**	**−0.09**	**0.029**	**−0.18 to −0.01**
	Partial closure	**−0.23**	**0.000**	**0.04 to 0.85**	**−0.14**	**0.002**	**−0.22 to −0.05**	**−**0.03	0.447	**−**0.10 to 0.05
	Partial outsourcing	**−0.12**	**0.009**	**0.82 to 0.97**	**−**0.02	0.679	**−**0.13 to 0.08	0.06	0.197	**−**0.06 to 0.15
Fair leadership	Reorganization	**−0.08**	**0.000**	**0.90 to 0.94**	**−0.05**	**0.000**	**−0.08 to −0.03**	**−**0.02	0.116	**−**0.04 to 0.01
	Downsizing	**−0.05**	**0.000**	**0.92 to 0.97**	**−0.04**	**0.009**	**−0.07 to 0.01**	**−**0.03	0.078	**−**0.05 to 0.00
	Layoffs	**−0.05**	**0.013**	**0.92 to 0.99**	**−0.07**	**0.004**	**−0.12 to −0.02**	**−0.06**	**0.010**	**−0.10 to −0.01**
	Partial closure	**−0.08**	**0.000**	**0.89 to 0.95**	**−0.09**	**0.000**	**−0.13 to −0.05**	**−0.05**	**0.009**	**−0.09 to −0.01**
	Partial outsourcing	**−0.04**	**0.005**	**0.92 to 0.99**	**−0.08**	**0.002**	**−0.13 to −0.03**	**−0.06**	**0.016**	**−0.10 to −0.01**
Job control decision	Reorganization	**−0.07**	**0.000**	**0.90 to 0.97**	**−0.06**	**0.006**	**−0.11 to −0.02**	**−**0.03	0.178	**−**0.06 to 0.01
	Downsizing	**−0.10**	**0.000**	**0.97 to 0.95**	**−0.07**	**0.005**	**−0.13 to −0.02**	**−**0.02	0.425	**−**0.06 to 0.03
	Layoffs	**−0.11**	**0.001**	**0.94 to 0.95**	**−0.100**	**0.014**	**−0.18 to −0.02**	**−**0.04	0.291	**−**0.11 to 0.03
	Partial closure	**−0.08**	**0.005**	**0.97 to 0.98**	0.01	0.784	**−**0.06 to 0.08	0.04	0.168	**−**0.02 to 0.10
	Partial outsourcing	**−**0.05	0.131	0.89 to 1.02	**−**0.01	0.752	**−**0.09 to 0.07	0.01	0.816	**−**0.06 to 0.08
Job demands quantitative	Reorganization	**0.16**	**0.000**	**1.13 to 1.22**	**0.19**	**0.000**	**0.14 to 0.24**	**0.06**	**0.019**	**0.01 to 0.10**
	Downsizing	**0.22**	**0.000**	**1.19 to 1.30**	**0.13**	**0.002**	**0.05 to 0.21**	0.04	0.289	**−**0.03 to 0.11
	Layoffs	**0.15**	**0.000**	**1.09 to 1.25**	**0.14**	**0.000**	**0.07 to 0.20**	**0.08**	**0.009**	**0.02 to 0.14**
	Partial closure	**0.09**	**0.002**	**1.04 to 1.16**	**0.12**	**0.007**	**0.03 to 0.20**	0.05	0.161	**−**0.02 to 0.12
	Partial outsourcing	**0.13**	**0.000**	**1.06 to 1.22**						
Role clarity	Reorganization	**−0.09**	**0.000**	**0.88 to 0.94**	**−0.07**	**0.000**	**−0.11 to −0.03**	**−**0.03	0.130	**−**0.06 to 0.01
	Downsizing	**−0.09**	**0.000**	**0.87 to 0.95**	**−0.09**	**0.000**	**−**0.14 to **−**0.05	**−0.05**	**0.026**	**−0.09 to −0.01**
	Layoffs	**−**0.05	0.075	0.90 to 1.01	**−0.07**	**0.074**	**−0.14 to 0.01**	**−**0.06	0.085	**−**0.13 to 0.01
	Partial closure	**−0.12**	**0.000**	**0.85 to 0.93**	**−0.13**	**0.000**	**−**0.19 to **−**0.06	**−0.07**	**0.019**	**−0.12 to −0.01**
	Partial outsourcing	**−0.07**	**0.033**	**0.88 to 0.99**	**−0.11**	**0.007**	**−0.19 to −0.03**	**−**0.07	0.065	**−**0.14 to 0.01
Role conflict	Reorganization	**0.24**	**0.000**	**1.23 to 1.33**	**0.22**	**0.000**	**0.18 to 0.26**	**0.10**	**0.000**	**0.06 to 0.13**
	Downsizing	**0.28**	**0.000**	**1.27 to 1.39**	**0.20**	**0.000**	**0.14 to 0.25**	0.04	0.076	**−**0.00 to 0.09
	Layoffs	**0.21**	**0.000**	**1.16 to 1.32**	**0.18**	**0.000**	**0.10 to 0.26**	**0.09**	**0.009**	**0.02 to 0.16**
	Partial closure	**0.24**	**0.000**	**1.20 to 1.35**	**0.17**	**0.000**	**0.11 to 0.24**	0.05	0.090	**−**0.00 to 0.11
	Partial Outsourcing	**0.12**	**0.001**	**1.05 to 1.21**	0.02	0.562	**−**0.06 to 0.11	**−**0.04	0.320	**−**0.11 to 0.04
Social climate	Reorganization	**−0.20**	**0.000**	**0.79 to 0.86**	**−0.20**	**0.000**	**−0.24 to −0.15**	**−0.09**	**0.000**	**−0.13 to −0.05**
	Downsizing	**−0.13**	**0.000**	**0.83 to 0.92**	**−0.13**	**0.000**	**−0.18 to −0.07**	**−**0.03	0.158	**−**0.08 to 0.01
	Layoffs	**−0.21**	**0.000**	**0.75 to 0.87**	**−0.20**	**0.000**	**−0.28 to −0.11**	**−0.09**	**0.018**	**−0.16 to −0.02**
	Partial closure	**−0.22**	**0.000**	**0.75 to 0.86**	**−0.21**	**0.000**	**−0.28 to −0.14**	**−0.09**	**0.005**	**−0.15 to −0.03**
	Partial outsourcing	**−0.23**	**0.000**	**0.73 to 0.86**	**−0.23**	**0.000**	**−0.31 to −0.14**	**−0.14**	**0.000**	**−0.22 to −0.06**
Support co-worker	Reorganization	**−0.08**	**0.000**	**0.89 to 0.95**	**−0.06**	**0.002**	**−0.10 to −0.02**	**−**0.02	0.173	**−**0.06 to 0.01
	Downsizing	**−0.10**	**0.000**	**0.98 to 0.94**	**−0.09**	**0.000**	**−0.13 to −0.04**	**−**0.04	0.072	**−**0.08 to 0.00
	Layoffs	**−0.11**	**0.000**	**0.85 to 0.95**	**−0.10**	**0.007**	**−0.17 to −0.03**	**−**0.05	0.097	**−**0.12 to 0.01
	Partial closure	**−0.14**	**0.000**	**0.83 to 0.91**	**−0.10**	**0.001**	**−0.17 to −0.04**	**−**0.04	0.151	**−**0.09 to 0.02
	Partial outsourcing	**−0.12**	**0.000**	**0.84 to 0.95**	**−0.09**	**0.020**	**−0.17 to −0.02**	**−**0.04	0.251	**−**0.11 to 0.03
Support superior	Reorganization	**−0.16**	**0.000**	**0.82 to 0.89**	**−0.13**	**0.000**	**−0.18 to −0.08**	**−**0.04	0.086	**−**0.09 to 0.01
	Downsizing	**−0.21**	**0.000**	**0.77 to 0.85**	**−0.14**	**0.000**	**−0.20 to −0.08**	**−**0.04	0.181	**−**0.09 to 0.02
	Layoffs	**−0.25**	**0.000**	**0.72 to 0.84**	**−0.18**	**0.000**	**−0.28 to −0.09**	**−**0.07	0.099	**−**0.15 to 0.01
	Partial closure	**−0.29**	**0.000**	**0.70 to 0.80**	**−0.17**	**0.000**	**−0.26 to −0.09**	**−**0.03	0.418	**−0.**11 to 0.04
	Partial outsourcing	**−0.16**	**0.000**	**0.79 to 0.93**	**−**0.11	0.040	**−**0.21 to **−**0.01	**−**0.01	0.799	**−**0.10 to 0.08
Predictability 1 month	Reorganization	**−0.18**	**0.000**	**0.81 to 0.86**	**−0.13**	**0.00**	**−0.17 to −0.09**	**−0.06**	**0.001**	**−0.09 to −0.02**
	Downsizing	**−0.15**	**0.000**	**0.83 to 0.90**	**−0.10**	**0.000**	**−0.15 to −0.06**	**−0.05**	**0.022**	**−0.10 to −0.01**
	Layoffs	**−0.19**	**0.000**	**0.78 to 0.88**	**−0.18**	**0.000**	**−0.26 to −0.10**	**−0.12**	**0.002**	**−0.19 to −0.04**
	Partial closure	**−0.20**	**0.000**	**0.78 to 0.86**	**−0.21**	**0.000**	**−0.28 to −0.15**	**−0.13**	**0.000**	**−0.19 to −0.07**
	Partial outsourcing	**−0.21**	**0.000**	**0.76 to 0.87**	**−0.19**	**0.000**	**−0.27 to −0.11**	**−0.12**	**0.001**	**−0.20 to −0.05**
Predictability 2 years	Reorganization	**−0.13**	**0.000**	**0.84 to 0.92**	**−0.11**	**0.000**	**−0.16 to −0.05**	**−0.06**	**0.023**	**−0.11 to 0.01**
	Downsizing	**−0.21**	**0.000**	**0.77 to 0.86**	**−0.13**	**0.000**	**−0.19 to −0.06**	**−0.06**	**0.047**	**−0.12 to −0.00**
	Layoffs	**−0.21**	**0.000**	**0.75 to 0.88**	**−0.15**	**0.003**	**−0.25 to −0.05**	**−0.10**	**0.021**	**−0.19 to −0.02**
	Partial closure	**−0.28**	**0.000**	**0.71 to 0.81**	**−0.16**	**0.000**	**−0.25 to −0.08**	**−**0.06	0.085	**−**0.14 to 0.01
	Partial outsourcing	**−0.30**	**0.000**	**0.68 to 0.81**	**−0.25**	**0.000**	**−0.35 to −0.14**	**−0.14**	**0.004**	**−0.23 to −0.04**

*Univariate analyses, “adjusted own effect”: cross-sectional and prospective analyses, Separate, linear regressions with separate, organizational change at baseline as predictor and work factor as outcome. Change < 12 months prior. Model I: adjusted for age, sex, skill level, and place of employment. Change > 24 months prior. Model I: adjusted for age, sex, skill level, and place of employment. Model II: adjusted for age, sex, skill level, place of employment, and work factor at baseline. The bold significance values are p < 0.05.*

##### Multivariate

The multivariate analyses showed fewer significant associations, however all work factors were significantly associated with at least one type of specific change each with *B-*values ranging from 0.20 to −0.17. See Table 5 for further details.

**TABLE 5:** Separate organizational change.

		**Change <12 months prior**			**Change >24 months prior**					
		**Model I**			**Model I**			**Model II**		
		***B***	***p*-Value**	**95% CI**	***B***	***p*-Value**	**95% CI**	***B***	***p*-Value**	**95% CI**
Empowering leadership	Reorganization	−0.05	0.108	0.90 to 1.01	−0.05	0.096	0.09 to 1.01	−0.01	0.660	0.94 to 1.039
	Downsizing	−0.02	0.562	0.91 to 1.05	−0.04	0.355	0.90 to 1.04	−0.00	0.960	−0.94 to 1.06
	Layoffs	−**0.12**	**0.023**	**0.79** to **0.98**	−**0.14**	**0.010**	**0.78** to **0.97**	−0.09	0.063	0.84 to 1.01
	Partial closure	−**0.11**	**0.021**	**0.81** to **0.98**	−0.09	0.069	0.83 to 1.01	−0.03	0.547	0.90 to 1.06
	Partial outsourcing	0.05	0.328	0.95 to 1.18	0.05	0.357	0.9 to 1.18	0.08	0.092	0.99 to 1.20
Fair leadership	Reorganization	−**0.04**	**0.009**	**0.94 to 0.99**	−**0.04**	**0.010**	**0.94 to 0.99**	−0.01	0.537	0.97 to 7.02
	Downsizing	−0.01	0.797	0.96 to 1.03	0.01	0.748	0.96 to 1.03	−0.01	0.707	0.96 to 1.03
	Layoffs	−0.03	0.291	0.93 to 1.02	−0.04	0.129	0.92 to 1.01	−0.04	0.085	0.92 to 1.01
	Partial closure	−**0.05**	**0.033**	**0.91 to 1.00**	−**0.05**	**0.024**	**0.91 to 0.99**	−0.02	0.240	0.94 to 1.02
	Partial outsourcing	−0.04	0.119	0.91 to 1.01	−0.05	0.081	0.91 to 0.1.01	−0.04	0.106	0.92 to 1.01
Job control decision	Reorganization	−**0.05**	**0.020**	**0.92 to 0.99**	−**0.05**	**0.035**	**0.91 to 0.99**	−0.03	0.199	0.94 to 1.01
	Downsizing	−**0.07**	**0.009**	**0.89 to 0.98**	−**0.07**	**0.018**	**0.88 to 0.99**	−0.02	0.324	0.93 to 1.02
	Layoffs	−0.05	0.144	0.88 to 1.02	−**0.08**	**0.057**	**0.85 to 1.00**	−0.04	0.228	0.98 to 1.03
	Partial closure	−0.02	0.550	0.92 to 1.05	0.06	0.117	0.99 to 1.15	0.06	0.064	1.00 to 1.13
	Partial outsourcing	0.01	0.857	0.94 to 1.09	0.01	0.747	0.93 to 1.10	0.01	0.769	0.94 to 1.09
Job demands quantitative	Reorganization	**0.13**	**0.000**	**1.09 to 1.19**	**0.12**	**0.000**	**1.10 to 1.18**	**0.05**	**0.008**	**1.01 to 1.10**
	Downsizing	**0.18**	**0.000**	**1.13 to 1.26**	**0.14**	**0.000**	**1.10 to 1.22**	0.02	0.355	0.97 to 1.08
	Layoffs	0.04	0.333	0.96 to 1.11	0.03	0.453	0.95 to 1.13	0.01	0.830	0.94 to 1.09
	Partial closure	−0.05	0.141	0.89 to 1.02	0.02	0.544	0.95 to 1.11	0.05	0.165	0.98 to 1.12
	Partial outsourcing	0.05	0.210	0.97 to 1.13	0.02	0.634	0.94 to 1.11	0.01	0.742	0.94 to 1.09
Role clarity	Reorganization	−**0.07**	**0.000**	**0.90 to 0.96**	−**0.04**	**0.038**	**0.92 to 0.99**	−0.01	0.660	0.9 to 1.03
	Downsizing	−**0.06**	**0.011**	**0.90 to 0.99**	−0.05	0.055	0.91 to 1.00	−0.02	0.365	0.94 to 1.02
	Layoffs	0.02	0.525	0.96 to 1.09	−0.02		0.93 to 1.08	−0.03	0.448	0.91 to 1.04
	Partial closure	−**0.06**	**0.041**	**0.89 to 0.100**	−**0.08**	**0.019**	**0.96 to 0.99**	−0.05	0.149	0.90 to 1.02
	Partial outsourcing	−0.01	0.820	0.93 to 1.06	−0.05	0.203	0.87 to 1.03	−0.04	0.277	0.89 to 1.03
Role conflict	Reorganization	**0.19**	**0.000**	**1.16 to 1.26**	**0.19**	**0.000**	**1.15 to 1.26**	**0.09**	**0.000**	**1.05 to 1.14**
	Downsizing	**0.20**	**0.000**	**1.16 to 1.28**	**0.12**	**0.000**	**1.06 to 1.20**	0.00	0.900	0.95 to 1.06
	Layoffs	0.07	0.058	1.00 to 1.150	**0.09**	**0.043**	**1.00 to 1.18**	**0.08**	**0.046**	**1.00 to 1.16**
	Partial closure	**0.09**	**0.006**	**1.03 to 1.17**	**0.08**	**0.045**	**1.00 to 1.16**	0.03	0.344	0.97 to 1.10
	Partial outsourcing	−0.04	0.246	0.89 to 1.03	−**0.11**	**0.012**	**0.82 to 0.98**	−**0.09**	**0.022**	**0.85 to 0.99**
Social climate	Reorganization	−**0.17**	**0.000**	**0.81 to 0.88**	−**0.16**	**0.000**	**0.82 to 0.89**	−**0.07**	**0.000**	**0.89 to 0.97**
	Downsizing	−**0.09**	**0.001**	**0.87 to 0.96**	−0.02	0.481	0.93 to 1.04	0.02	0.471	0.97 to 1.07
	Layoffs	−**0.13**	**0.000**	**0.82 to 0.95**	−**0.11**	**0.009**	**0.82 to 0.97**	−0.06	0.122	0.87 to 1.02
	Partial closure	−**0.15**	**0.000**	**0.81 to 0.82**	−**0.11**	**0.006**	**0.93 to 0.97**	−0.04	0.211	0.89 to 1.03
	Partial outsourcing	−0.03	0.482	0.91 to 1.05	−**0.12**	**0.013**	**0.81 to 0.98**	−**0.10**	**0.016**	**0.84 to 0.98**
Support co-worker	Reorganization	−**0.05**	**0.007**	**0.81 to 0.99**	−0.04	0.054	0.92 to 1.00	−0.02	0.328	0.95 to 1.02
	Downsizing	−0.04	0.102	0.92 to 1.01	−0.04	0.099	0.91 to 1.01	−0.02	0.388	0.94 to 1.03
	Layoffs	−0.05	0.091	0.89 to 1.01	−0.05	0.208	0.88 to 1.03	−0.03	0.371	0.91 to 1.04
	Partial closure	−**0.07**	**0.020**	**0.88 to 0.99**	−0.06	0.084	0.88 to 1.01	−0.03	0.416	0.92 to 1.04
	Partial outsourcing	−0.05	0.132	0.89 to 1.02	−0.04	0.388	0.89 to 1.05	−0.01	0.779	0.92 to 1.06
Support superior	Reorganization	−**0.09**	**0.000**	**0.87 to 0.96**	−**0.09**	**0.001**	**0.86 to 0.96**	−0.03	0.155	0.92 to 1.01
	Downsizing	−**0.11**	**0.000**	**0.85 to 0.95**	−**0.07**	**0.037**	**0.87 to 1.00**	−0.02	0.508	0.92 to 1.04
	Layoffs	−**0.14**	**0.001**	**0.81 to 0.95**	−**0.11**	**0.041**	**0.83 to 0.99**	−0.05	0.251	0.87 to 1.04
	Partial closure	−**0.17**	**0.000**	**0.78 to 0.91**	−**0.10**	**0.036**	**0.93 to 0.99**	−0.01	0.745	0.91 to 1.07
	Partial outsourcing	0.00	0.959	0.92 to 1.09	−0.00	0.983	0.90 to 1.11	0.02	0.687	0.93 to 1.18
Predictability 1 month	Reorganization	−**0.14**	**0.000**	**0.83 to 0.90**	−**0.09**	**0.000**	**0.88 to 0.95**	−0.04	0.056	0.93 to 1.00
	Downsizing	−**0.07**	**0.005**	**0.89 to 0.98**	−0.01	0.605	0.93 to 1.04	0.00	0.967	0.95 to 1.05
	Layoffs	−**0.09**	**0.011**	**0.86 to 0.98**	−**0.10**	**0.021**	**0.93 to 0.99**	−0.07	0.066	0.86 to 1.01
	Partial closure	−**0.08**	**0.012**	**0.87 to 0.98**	−**0.14**	**0.000**	**0.81 to 0.94**	−**0.09**	**0.009**	**0.85 to 0.98**
	Partial outsourcing	−**0.10**	**0.004**	**0.84 to 0.97**	−**0.09**	**0.041**	**0.84 to 0.99**	−0.06	0.133	0.87 to 1.02
Predictability 2 years	Reorganization	−**0.07**	**0.003**	**0.88 to 0.98**	−**0.07**	**0.022**	**0.88 to 0.99**	−0.04	0.179	0.92 to 1.02
	Downsizing	−**0.11**	**0.000**	**0.84 to 0.95**	−0.05	0.141	0.88 to 1.02	−0.02	0.561	0.92 to 1.05
	Layoffs	−0.08	0.080	0.85 to 1.01	−0.06	0.230	0.85 to 1.04	−0.06	0.191	0.86 to 1.03
	Partial closure	−**0.16**	**0.000**	**0.79 to 0.93**	−0.06	0.215	0.96 to 1.04	−0.00	0.981	0.92 to 1.08
	Partial outsourcing	−**0.17**	**0.000**	**0.77 to 0.92**	−**0.18**	**0.001**	**0.75 to 0.93**	−**0.11**	**0.024**	**0.81 to 0.99**

*Multivariate analyses: cross-sectional and prospective analyses. Separate, multivariable linear regressions with work factor as outcome and specific change at baseline as predictor, mutually adjusted for each other. Change < 12 months prior. Model I: adjusted for age, sex, skill level, and place of employment. Change > 24 months prior. Model I: adjusted for age, sex, skill level, and place of employment. Model II: adjusted for age, sex, skill level, place of employment, and work factor at baseline. The bold significance values are p < 0.05.*

#### Prospective Analyses

##### Univariate

The univariate analyses pertaining to the effect of change reported to have taken place more than 24 months prior showed all types of specific changes to be associated with at least three work factors (*B-*values ranging −0.14 to 0.05 in Model I. In Model II, i.e., also adjusted for baseline levels of each respective work factor, fewer associations remained statistically significant. For further details see Table 4.

##### Multivariate

In the multivariate analyses, most significant associations were no longer statistically significant, although empowering leadership, job demands, role conflict, social climate, job predictability, and future employability remained significantly associated with certain types of specific change. See Table 5 for further details.

### Effects of Multiple Organizational Changes

#### Cross-Sectional Analyses

Separate linear regressions for each work factor as outcome showed exposure to more than one type of change 12 months prior to be statistically significantly associated with all work factors (*b-*values ranging from 0.27 to −0.26).

#### Prospective Analyses

Separate linear regressions with each work factor as outcome showed exposure to more than one type of change 24 months prior to be statistically significantly associated with all work factors (*b-*values ranging from 0.27 to −0.07, see Table 2) in Model I. When controlled for baseline levels of the respective work factor, Model II, statistically significant associations were seen in seven of the work factors (*b-*values ranging from −0.11 to −0.03). See Table 6 for further details.

**TABLE 6:** Multiple organizational changes (at baseline).

		**Change <12 months prior**			**Change >24 months prior**					
		**Model I**			**Model I**			**Model II**		
		***B***	***p*-Value**	**95% CI**	***B***	***p*-Value**	**95% CI**	***B***	***p*-Value**	**95% CI**
Empowering leadership	No change at T1	–	–	–	–	–	–	–	–	–
	One change at T1	−0.03	0.329	0.91 to 1.03	−0.03	0.362	−0.10 to 0.04	−0.01	0.854	−0.06 to 0.05
	Two or more changes at T1	−**0.12**	**0.001**	**0.83 to 0.95**	−**0.12**	**0.001**	−0.19 to −0.05	−0.02	0.545	−0.08 to 0.04
Fair leadership	No change at T1	–	–	–	–	–	–	–	–	–
	One change at T1	−0.02	0.145	0.95 to 1.01	−0.02	0.158	−0.05 to 0.01	−0.00	0.918	−0.03 to 0.03
	Two or more changes at T1	−**0.07**	**0.000**	**0.91 to 0.97**	**0.07**	**0.000**	−**0.10 to**−**0.04**	−**0.03**	**0.036**	−**0.07 to**−**0.00**
Job demands quantitative	No change at T1	–	–	–	–	–	–	–	–	–
	One change at T1	**0.14**	**0.000**	**1.09 to 1.21**	**0.14**	**0.000**	**0.09 to 0.20**	**0.07**	**0.003**	**0.02 to 0.11**
	Two or more changes at T1	**0.25**	**0.000**	**1.21 to 1.36**	**0.25**	**0.000**	**0.19 to 0.31**	**0.10**	**0.000**	**0.18 to 0.28**
Job control decision	No change at T1	–	–	–	–	–	–	–	–	–
	One change at T1	−0.05	0.059	0.90 to 1.00	−0.04	0.185	−0.09 to 0.02	**−**0.03	0.202	**−**0.07 to 0.02
	Two or more changes at T1	−**0.09**	**0.002**	**0.86 to 0.97**	−**0.08**	**0.007**	−**0.14 to**−**0.02**	−0.02	0.399	−0.07 to 0.03
Role clarity	No change at T1	–	–	–	–	–	–	–	–	–
	One change at T1	−0.02	0.448	0.94 to 1.03	−0.02	0.403	−0.07 to 0.03	**−**0.00	0.838	**−**0.04 to 0.04
	Two or more changes at T1	−**0.112**	**0.000**	**0.84 to 0.94**	−**0.12**	**0.000**	−**0.17 to**−**0.07**	−**0.06**	**0.015**	−**0.10 to**−**0.01**
Role conflict	No change at T1	–	–	–	–	–	–	–	–	–
	One change at T1	**0.16**	**0.000**	**1.11 to 1.24**	**0.16**	**0.000**	**0.11 to 0.22**	**0.08**	**0.001**	**0.03 to 0.12**
	Two or more changes at T1	**0.27**	**0.000**	**1.24 to 1.39**	**0.27**	**0.000**	**0.21 to 0.33**	**0.08**	**0.003**	**0.03 to 0.13**
Social climate	No change at T1	–	–	–	–	–	–	–	–	–
	One change at T1	−**0.10**	**0.000**	**0.86 to 0.95**	−**0.11**	**0.000**	−**0.16 to**−**0.05**	−0.04	0.078	−0.09 to 0.01
	Two or more changes at T1	−**0.26**	**0.000**	**0.73 to 0.82**	−**0.26**	**0.000**	−**0.31 to**−**0.20**	−**0.10**	**0.000**	−**0.16 to**−**0.05**
Support co-worker	No change at T1	–	–	–	–	–	–	–	–	–
	One change at T1	−0.01	0.692	0.95 to 1.04	−0.01	0.736	−0.05 to 0.04	0.01	0.764	−0.03 to 0.05
	Two or more changes at T1	−**0.09**	**0.000**	**0.86 to 0.96**	−**0.10**	**0.000**	−**0.15 to 0.04**	−0.05	0.052	−0.09 to 0.00
Support superior	No change at T1	–	–	–	–	–	–	–	–	–
	One change at T1	−**0.07**	**0.019**	**0.87 to 0.99**	−**0.07**	**0.033**	−**0.13 to**−**0.01**	−0.04	0.207	−0.09 to 0.02
	Two or more changes at T1	−**0.19**	**0.000**	**0.77 to 0.89**	−**0.19**	**0.000**	−**0.26 to**−**0.12**	−0.05	0.096	−0.11 to 0.01
Predictability 1 month	No change at T1	–	–	–	–	-	-	-	-	-
	One change at T1	−**0.07**	**0.003**	**0.89 to 0.98**	−**0.07**	**0.003**	−**0.11 to**−**0.02**	−0.03	0.124	−0.07 to 0.01
	Two or more changes at T1	−**0.21**	**0.000**	**0.77 to 0.85**	−**0.22**	**0.000**	−**0.27 to**−**0.16**	−**0.11**	**0.000**	−**0.16 to**−**0.06**
Predictability 2 years	No change at T1	–	–	–	–	–	–	–	–	–
	One change at T1	−0.05	0.109	0.89 to 1.01	−0.06	0.098	−0.12 to 0.01	−0.04	0.251	−0.10 to 0.03
	Two or more changes at T1	−**0.19**	**0.000**	**0.77 to 0.89**	−0.19	**0.000**	−**0.26 to**−**0.12**	−**0.10**	**0.002**	−**0.17 to**−**0.04**

*Cross-sectional and prospective analyses. Separate, linear regressions with frequency of change at baseline as predictor and work factor as outcome. Change < 12 months prior. Model I: adjusted for age, sex, skill level, and place of employment. Change > 24 months prior. Model I: adjusted for age, sex, skill level, and place of employment. Model II: adjusted for age, sex, skill level, place of employment, and work factor at baseline. The bold significance values are p < 0.05.*

### Effects of Repeated Organizational Change

Separate linear regressions were run with each work factor as the outcome. In Model I, repeated, organizational change was statistically significantly associated with all work factors, with *b-*values ranging from 0.39 to −0.04. In Model II (controlling for baseline levels of the respective work factor) associations remained statistically significant, with *b-*values ranging from −19 to 0.18. See Table 7 for further details.

**TABLE 7:** Repeated organizational change.

		**Model I**			**Model II**		
		***B***	***p***	**95% CI**	***B***	***p***	**95% CI**
Empowering leadership	No change at T1 or T2	–	–	–	–	–	–
	At least one change at T1 only	−0.08	0.064	−0.17 to 0.01	−0.02	0.536	−0.10 to 0.05
	At least one change at T2 only	−**0.15**	**0.002**	−**0.05** to −**0.06**	−**0.13**	**0.003**	−**0.21** to −**0.04**
	At least one change at *both* T1 T2	−**0.16**	**0.000**	−**0.24** to −**0.08**	−**0.08**	**0.013**	−**0.15** to −**0.02**
Fair leadership	No change at T1 or T2	–	–	–	–	–	–
	At least one change at T1 only	−**0.04**	**0.026**	−**0.08** to −**0.01**	−0.02	0.430	−0.05 to 0.02
	At least one change at T2 only	−**0.09**	**0.000**	−**0.14** to −**0.05**	−**0.07**	**0.001**	−**0.11** to −**0.03**
	At least one change at *both* T1 T2	−**0.11**	**0.000**	−**0.14** to −**0.07**	−**0.06**	**0.000**	−**0.10** to −**0.03**
Job demands quantitative	No change at T1 or T2	–	–	–	–	–	–
	At least one change at T1 only	**0.19**	**0.000**	**0.12** to **0.26**	**0.08**	**0.005**	**0.23** to **0.14**
	At least one change at T2 only	**0.17**	**0.000**	**0.10** to **0.26**	**0.10**	**0.001**	**0.04** to **0.17**
	At least one change at *both* T1 T2	**0.31**	**0.000**	**0.24** to **0.37**	**0.15**	**0.000**	**0.10** to **0.20**
Job control decision	No change at T1 or T2	–	–	–	–	–	–
	At least one change at T1 only	−0.03	0.348	−0.10 to 0.04	0.02	0.515	−0.08 to 0.40
	At least one change at T2 only	−**0.10**	**0.006**	−**0.19** to **0.03**	−**0.08**	**0.019**	−**0.14** to −**0.01**
	At least one change at *both* T1 T2	−**0.15**	**0.000**	−**0.21** to −**0.09**	−**0.10**	**0.000**	−**0.15** to −**0.04**
Role clarity	No change at T1 or T2	–	–	–	–	–	–
	At least one change at T1 only	−0.05	0.087	−0.11 to 0.01	−0.03	0.236	0.08 to 0.02
	At least one change at T2 only	−**0.11**	**0.002**	−**0.17** to −**0.04**	−**0.11**	**0.000**	−**0.16** to −**0.05**
	At least one change at *both* T1 T2	−**0.13**	**0.000**	−**0.19** to −**0.08**	−**0.09**	**0.000**	−**0.14** to −**0.05**
Role conflict	No change at T1 or T2	–	–	–	–	–	–
	At least one change at T1 only	**0.14**	**0.000**	**0.07** to **0.21**	0.02	0.464	−0.04 to 0.08
	At least one change at T2 only	**0.20**	**0.000**	**0.13** to **0.28**	**0.10**	**0.006**	**0.03** to **0.16**
	At least one change at *both* T1 T2	**0.39**	**0.000**	−**0.33** to **0.45**	**0.18**	**0.000**	**0.12** to **0.24**
Social climate	No change at T1 or T2	–	–	–	–	–	–
	At least one change at T1 only	−**0.14**	**0.000**	−**0.21** to −**0.08**	−0.06	0.059	−0.11 to 0.00
	At least one change at T2 only	−**0.21**	**0.000**	−**0.28** to −**0.14**	−**0.16**	**0.000**	−**0.23** to −**0.10**
	At least one change at *both* T1 T2	−**0.34**	**0.000**	−**0.40** to −**0.28**	−**0.19**	**0.000**	−**0.24** to −**0.13**
Support co-worker	No change at T1 or T2	–	–	–	–	–	–
	At least one change at T1 only	−0.03	0.343	−0.09 to 0.03	0.01	0.800	−0.05 to 0.06
	At least one change at T2 only	−**0.12**	**0.001**	−**0.19** to −**0.05**	−**0.09**	**0.004**	−**0.15** to −**0.03**
	At least one change at *both* T1 T2	−**0.13**	**0.000**	−**0.18** to −**0.08**	−**0.08**	**0.001**	−**0.13** to **0.03**
Support superior	No change at T1 or T2	–	–	–	–	–	–
	At least one change at T1 only	−**0.11**	**0.006**	−**0.19** to −**0.03**	−0.04	0.322	−0.10 to 0.03
	At least one change at T2 only	−**0.22**	**0.000**	−**0.31** to −**0.13**	−**0.17**	**0.000**	−**0.24** to −**0.09**
	At least one change at *both* T1 T2	−**0.27**	**0.000**	−**0.34** to −**0.20**	−**0.16**	**0.000**	−**0.22** to −**0.09**
Predictability 1 month	No change at T1 or T2	–	–	–	–	–	–
	At least one change at T1 only	−**0.06**	**0.033**	−**0.12** to −**0.01**	−0.01	0.741	−0.06 to 0.04
	At least one change at T2 only	−**0.16**	**0.000**	−**0.22** to −**0.09**	−**0.11**	**0.000**	−**0.17** to −**0.06**
	At least one change at *both* T1 T2	−**0.27**	**0.000**	−**0.32** to −**0.22**	−**0.17**	**0.000**	−**0.22** to −**0.12**
Predictability 2 years	At least one change at T1 only	−0.08	0.063	−0.17 to 0.00	−0.06	0.123	−0.14 to 0.02
	At least one change at T2 only	−**0.12**	**0.017**	−**0.22** to −**0.02**	−**0.13**	**0.004**	−**0.22** to −**0.04**
	At least one change at *both* T1 T2	−**0.21**	**0.000**	−**0.29** to −**0.13**	−**0.16**	**0.000**	−**0.23** to −**0.08**

*Separate, linear regressions with repeated change at baseline as predictor and work factor at follow-up as outcome. Model I: adjusted for age, sex, skill level, and place of employment. Model II: adjusted for age, sex, skill level, place of employment, and work factor at baseline. The bold significance values are p < 0.05.*

## Discussion

### Effects of Separate Organizational Change on the Work Environment

The present study demonstrated statistically significant cross-sectional and prospective relationships between various discrete types of organizational change and a number of specific work factors. Hence, changes in multiple work factors were demonstrated when the organizational change had taken place within the last 12 months and more than 24 months prior. In the prospective analyses most associations were no longer significant, which may indicate that for some work factors, the adverse impact of organizational changes are primarily manifested more proximal to the change.

In light of the present results, organizational change seems to have both a short-term and a long-term effect on multiple factors in the work environment. The short-term effect seems to emerge during and be manifest shortly after change implementation, but then diminish over time. For instance, the present results indicate that shortly after a company restructuring process, employees are more likely to report lower role clarity, i.e., more uncertainties regarding their job’s objectives and responsibilities, potentially due to the new job situation or tasks given because of the restructuring process. However, as time passes and employees get more conversant with their new role and responsibilities, the feeling of role clarity seems to increase. In other words, although the present study cannot point to why, the results show that the adverse effect of restructuring on perceived role clarity diminishes over time. In addition to a short-term effect, long-term effects of change on certain work factors were also shown in the present study. These long-term effects may also emerge during or shortly after change has taken place, but then stabilize and last long-term or continue to develop over time. For instance, the present results show that following a layoff process, employee’s perception of their superior as fair or empowering is affected proximal to the change, but also remains low 2 years after the layoff has taken place. An inevitable consequence of a layoff process it the termination of job contracts. Both the process and result of deciding who will be let off may give rise to the feeling of powerlessness, injustice and unfair treatment by superiors and management (Campbell-Jamison et al., 2001). One could surmise that this perception might dwindle over time for the remaining, or “surviving” employees, however, the present results indicate that this may not be the case, as employee perception of fair and empowering leadership continues to be low years after the implementation, even for those who are fortunate to keep their job and remain within the company (Vahtera et al., 2004). These results are in line with prior studies showing survivors of downsizing, layoffs, and outsourcing processes to report a lower sense of job security, productivity, organizational attachment, perceived organizational justice and higher turnover intention (Maertz et al., 2010; Drzensky and Heinz, 2015; van Dick et al., 2016).

To summarize, one interpretation of the pattern of associations observed in the present study could be that the adverse effects of organizational changes on the psychosocial work factors took place immediately or shortly after the change process. Moreover, while the effect diminished over time for most factors, the adverse effects remained or continued to unfold during the study’s timeframe for others. In the following, a brief discussion of the results pertaining to each, respective work factors is presented.

### Job Tasks (Job Demands and Job Control)

The present results demonstrated how employees perceived job demands to increase both short- and long-term following the implementation of various types of organizational change. In addition, a short-term effect was also observed for job control, with employees reporting less control following all included types of organizational change. These results are in line with prior studies reporting increased demands and lowered control following organizational changes such as restructuring and downsizing (Head et al., 2006; Egan et al., 2007; Tvedt et al., 2009). When implementing large-scaled change, job demands may increase due to change-related tasks, which comes in addition to ordinary tasks and responsibilities. Hence, the total workload may increase both while the process is running and more permanently (Kivimäki et al., 2001). One may speculate that the additional workload associated with change implementation may lower the feeling of job control and leave less resources for co-workers and superiors to be supportive during and following change. The Job Demand-Control (-Support) Model (JDCS) (Karasek and Theorell, 1990; Luchman and González-Morales, 2013) posits that the combination of high demands, low control and lack of support constitutes a high strain work environment, which may influence various employee outcomes, such as mental and somatic health, job satisfaction, turnover intention and productivity (Karasek, 1998; Bordia et al., 2004; Virtanen et al., 2006; Magnusson Hanson et al., 2008; van den Berg et al., 2008; Eller et al., 2009). However, high control and support may buffer the adverse effects of high demands; hence, facilitating a supportive social climate and help employees gain control over the new situation may be particularly important to buffer the adverse effects of the high job demands associated with extensive workplace changes (Van der Doef and Maes, 1999; Levi, 2000; Campbell-Jamison et al., 2001).

### Job Roles (Role Conflict and Role Clarity)

The present results also demonstrated reduced role clarity and increased role conflict, both short- and long-term, following various organizational changes. These results are in line with prior studies linking, for instance, exposure to company restructuring to an increase in role conflict and decrease in employees’ experience of clarity regarding their own tasks and responsibilities (Baillien and De Witte, 2009; Oreg et al., 2011). The heightened role conflict following organizational changes may potentially stem from additional or changed job demands without a corresponding adjustment of resource availability during or following changes (Oreg et al., 2011) or difficulties maintaining clearly defined goals and responsibilities for the individual worker at all times during an extensive change process. A restructuring process often involves redefining and rearranging employee tasks and responsibilities, and in the midst of the change-process, it may be challenging to design these explicitly to ensure that new demands are not in conflict with established ones. As prior meta-analyses have shown role conflict and –uncertainty to be related to employee health complaints (Stansfeld and Candy, 2006; Schmidt et al., 2014), it seems crucial to ensure role clarity and prevent role conflict during the process of extensive change.

### Leadership (Fair-, Empowering-, and Supportive Leadership)

The present results show that employees perceive their superiors as less fair and empowering following various types of organizational change, both short- and long-term. In addition, support from superior was also perceived to be lower following the included organizational changes, but effects were only present short-term. Various factors may influence employees’ perception of leadership during and following organizational changes. Implementing extensive change may, for instance, put increased pressure and workload on management and superiors, leaving fewer resources to preserve a sense of inclusive, supportive, and fair leadership style (Hoag et al., 2002). The need to make unpopular decisions may also affect how employees perceive management to be fair, empowering or supportive during or following the change process, especially if the process does not follow pre-existing guidelines and expectations (Tyler and De Cremer, 2005). The extent to which management includes employee concerns and perspectives in the process, as well as how management communicates the change have also been reported to affect how superiors and leadership are perceived both prior to, during and following changes (Hoag et al., 2002; Riolli and Savicki, 2006). Prior studies have demonstrated the importance of employee perceptions of organizational justice during organizational changes (Virtanen and Elovainio, 2018). The perception of low fairness from management has been associated with poor social climate and reduced productivity (Schyns and Schilling, 2013; Virtanen and Elovainio, 2018), as well as long-term and reoccurring sick leave, mental distress and somatic health complaints (Tyler and De Cremer, 2005; Riolli and Savicki, 2006; Meierhans et al., 2008; Robbins et al., 2012; Leineweber et al., 2017). On the other hand, employees who perceive leaders to act procedurally fair during organizational changes are more accepting of the change and view management and leaders as more competent and trustworthy in handling the change (Tyler and De Cremer, 2005). The present results showing how leaders are rated as less fair and empowering following organizational change processes may be of interest when planning change, as counteracting these effects may improve both the process, consequences and results of extensive workplace changes.

### Social Relations (Support From Co-workers and Social Climate)

Implementing organizational changes may also influence various aspects of an organization’s social and relational environment. The present results show both social climate and perceived support from co-workers to be lower following various types of change, although long-term effects were only shown for social climate. Various aspects of change implementation may affect social relations within the organization. Rearranging collegial composition, i.e., losing and/or being introduced to new co-workers, competing for the same positions during a restructuring process or getting a new superior, may all influence an organization’s social cohesion or employees ability to provide others with the support they normally are able to. The current results agree with prior studies reporting increased conflict, demoralization and reduced support following organizational change (Campbell and Pepper, 2007). Support and social climate have both been linked to employee health, productivity and (Magnusson Hanson et al., 2008; Ljungblad et al., 2014; Charoensukmongkol et al., 2016; Yang et al., 2016; Geldart et al., 2018) which makes focusing on the effects of change on the organization’s social relations an important aspect to consider in order to secure a healthy and successful change process.

### Job Predictability (Short-Term Job Predictability and Future Employability)

When implementing large-scaled organizational changes, it is naturally challenging to know how both the process and end-result will turn out. Thus, organizational changes are naturally associated with a certain degree of uncertainty. The present study showed employees’ sense of job predictability and future employability to be lower following various specific company changes both short- and long-term. Similar results have been reported in previous studies (Kivimäki et al., 2001; Probst, 2003; Baillien and De Witte, 2009). Reduced job predictability, i.e., attenuated ability to form reasonable expectations about the future, regarding both short-term job characteristics and long-term employment prospects, is intrinsically linked to the concept of job insecurity, which has been linked to employee outcomes such as somatic and mental health complaints (Hellgren et al., 1999; Ferrie, 2001; Ferrie et al., 2002; Staufenbiel and König, 2010; Landsbergis et al., 2014; De Witte et al., 2016), lowered efficiency, reduced organizational citizenship behavior and higher turnover intention (Hellgren et al., 1999; Probst, 2003; Staufenbiel and König, 2010). It may not be surprising that job predictability is temporarily affected by an extensive change process, however, the current results also indicate that the reduction in job predictability persists long after change implementation is completed. Prior studies have also shown that even though the cause of job insecurity was removed, the insecurity did not completely vanish (Ferrie et al., 2002), indicating that the sense of uncertainty may persist for longer periods. Furthermore, this lowered predictability does not only pertain to one’s current job, but also future job prospects, e.g., perceived future employability. Long-term effects on perceived job security have also been reported in prior studies (Ferrie et al., 1998; Probst, 2003). Such long-term effects on perceptions of predictability may be due to reduced trust in management and breaches in the implicit psychological contract in the workplace (Morgan and Zeffane, 2003). Reduced trust in management and perceived breaches in the psychological contract have been linked to various types of organizational change (Turnley and Feldman, 1998; Bellou, 2006). Although reduced predictability may be a common, proximal consequence of organizational change, it does not follow that it is a natural lasting consequence of change. Characteristics of change processes may influence the perception of unpredictability. For instance, prior studies have linked the extent to which employees are involved in the change process, e.g., employee participation in planning and implementing changes, with lower levels of uncertainty and higher levels of perceived control (Bordia et al., 2004). These results have been supported by prior studies reporting employee participation to be linked with higher perceived control, lower levels of job insecurity and reduced mental health complaints and sick leave (Bond and Bunce, 2001; Abildgaard et al., 2018). Results from the present study showed both short- and long-term adverse effects on job predictability and perceived future employability following organizational change. In light of the aforementioned studies linking both detrimental effects on both health and productivity with such uncertainty (Ferrie, 2001; Ferrie et al., 2002; Hellgren and Sverke, 2003; Staufenbiel and König, 2010; Landsbergis et al., 2014; De Witte et al., 2016), it seems imperative to keep job uncertainty to a minimum during and following change, possibly through involving employees in the process and by promoting a sense of control and support (Bordia et al., 2004).

### Effects of Multiple or Repeated Organizational Changes on Factors in the Work Environment

The present study demonstrated a stronger adverse effect on the majority of work factors following multiple changes at one time point compared to one specific change only. Furthermore, following repeated organizational changes, the adverse effects were stronger for all included work factors. These results are in line with prior studies reporting stronger effects following exposure to multiple or repeated organizational changes in work factors, such as role conflict and -ambiguity, social support, job insecurity, job demands, trust in management, and turnover intention (Ferrie et al., 2002; Isaksson et al., 2002; Moore et al., 2004; Wagstaff et al., 2016). Prior studies have also reported stronger effects following repeated change on various somatic and mental health complaints (Isaksson et al., 2002; Moore et al., 2004; Oreg et al., 2011). Although it remains uncertain why, these results indicate a cumulative effect of organizational change events on multiple aspects in the work environment, suggesting that organizations and its employees do not adapt to or assimilate to the new situation to the extent that the impact of change dissipates. Stress-vulnerability models may help explain the stronger effects on both perceptions of the work environment and health following repeated organizational changes (Zapf et al., 1996). These models posit that repeated exposure to a stressor may wear out an individual’s coping resources, which over time may lead to fatigue and weaken the ability to cope when re-exposed to the stressor. Hence, the stronger effects on employee perception of various aspect of the work environment following repeated company changes could be a result of fatigue and reduced resources to cope with change as exposure to prior changes have worn-out coping resources. As both prior and the present results show organizational changes to be associated with adverse effects on multiple factors in the psychosocial work environment known to influence both employee health and productivity, it seems imperative for organizations to prevent these unfavorable effects when planning and implementing change in order to secure both employee health and company sustainability. As the rate of change is increasing in contemporary work life a larger proportion of the workforce is likely to be exposed to organizational changes more than once during their career. As more employees will be facing multiple, large-scaled workplace changes, a focus on the prevention of the adverse effects associated with such changes seems vital.

### Methodological Considerations

Certain methodological limitations may have affected the generalizability of the present results. Regarding attrition, response rate at baseline was 82%, while 58% participated at follow up. Dropout was associated with being employed in the private sector, working in professions requiring 13–15, 10–12, and less than 10 years of formal education respectively. Hence, selection bias may have affected the external validity and by that compromised the generalizability of the present results.

The participating companies primarily contacted STAMI after an invitation to participate in the project was posted on the institute’s web pages, hence sampling was not random. Management was not asked about their reasons for participation. It may be that the organizations who contacted STAMI in order to participate, constitute a subset of Norwegian companies especially focused on the subject, perhaps more so than the average Norwegian firm. Furthermore, a larger part of the respondents was holding permanent positions and were employed in public sector compared to the general working population in Norway (Nergaard, 2016). Prior studies have shown temporary employees to report higher physical workload (Virtanen et al., 2006) and higher levels of mental health complaints (Aronsson et al., 2002; Virtanen et al., 2005, 2011). One may speculate whether employees not holding permanent positions experience extensive company changes as more of a threat to for instance their job security than those permanently employed. Due to the sample composition, the present results may underestimate the impact of organizational change on certain aspects of the work environment.

All data were collected by questionnaires; hence, both self-report bias and common-method bias could influence responses (Moorman and Podsakoff, 1992; Donaldson and Grant-Vallone, 2002). Precautions were taken in order to minimize such effects, e.g., a temporal separation of measurements, forced-choice items (Nederhof, 1985), baseline adjustments for all outcome variables and differences in wording and response options for predictor and outcome may all reduce the risk of common-method variance (Podsakoff et al., 2003). However, one cannot rule of the potential effects, the study had a prospective design with a 2-year interval between baseline and follow-up. This may not be the optimal interval to measure the effects of changes on the psychosocial work environment, with short-term effects possibly being present, but diminishing in the years between baseline and follow-up (Oreg et al., 2011). For this reason, the present study included both cross-sectional and prospective analyses. Keeping in mind the limitations in cross-sectional design regarding inference of causation (Levin, 2006), we chose to include these analyses due to items pertaining to change inquired into change taking place up to 12 months prior, while items related to work factors considered the perception of the employee’s current work environment. In addition, the risk of reverse causation in the relationship between organizational change and work factors in the study is considered small as extensive company changes are likely to be events whose occurrence and frequency to a lesser degree is affected by employee’s perception of specific psychological and social work factors.

### Future Perspectives

The present study elucidates the negative effects of exposure to separate and repeated organizational changes on employee’s perception of multiple aspects of the organization’s psychosocial work environment. In order to implement organizational changes in a healthy and successful manner, securing a healthy and productive work environment is crucial. Prior studies have indicated that participation in decision-making process (Egan et al., 2007), adhering to pre-existing guidelines (Korsgaard et al., 2002) and provide adequate and effective information and communication flow (Allen et al., 2007; Rehman, 2011) may influence employee health, attitudes and behaviors during and following organizational changes. Hence, organizations may potentially alleviate the effects of change by following pre-defined procedural and ethical guidelines and include employees in the change process. The present results highlight the work factors most susceptible to adverse effects following extensive workplace changes. An assessment of the underlying mechanisms in these relationships, explaining why and how different types of organizational changes affect the various psychosocial work factors differently, were outside the scope of the present study. To gain a further understanding of why and how implementing organizational change influence the various aspects of the work environment, further studies are needed to elucidate the specific mechanisms in order to obtain a more thorough understanding of these unique relationships. Preventing negative effects in psychosocial work factors should be a pivotal part of both change planning and implementation, as an unfavorable psychosocial work environment has been associated with adverse effects on both employee and company outcomes, such as health, turnover intention, productivity and profitability.

## Data Availability Statement

The datasets generated for this study are available on request to the corresponding author.

## Ethics Statement

This study was carried out in accordance with the recommendations of the Data Inspectorate of Norway and the Regional Committee for Medical and Health Research Ethics (REK), with written informed consent from all subjects. All subjects gave written informed consent in accordance with the Declaration of Helsinki. The protocol was approved by the Regional Committee for Medical and Health Research Ethics (REK).

## Author Contributions

LF participated in the idea development, conducted the analyses, and was responsible for writing the manuscript. JC participated in the data collection and idea development, contributed to the content, and read all versions of the manuscript. SK was responsible for the data collection and initiation of the project, participated in the idea development, contributed to structure and content, and read all versions of the manuscript.

## Conflict of Interest

The authors declare that the research was conducted in the absence of any commercial or financial relationships that could be construed as a potential conflict of interest.
